# Supplementary material for: The intensity of male-male competition may affect chemical scent constituents in the dark ventral patch of male Iberian red deer
Source: PLoS One. 2019 Sep 3;14(9):e0221980. doi: 10.1371/journal.pone.0221980 (PMC6719829; doi:10.1371/journal.pone.0221980)
Supplement: Attachment — Submitted filename: answer_plosone-def.docx [file pone.0221980.s001.docx]

**Answers to associated-editor comments**

1. We note that you have indicated that data from this study are available upon request. PLOS only allows data to be available upon request if there are legal or ethical restrictions on sharing data publicly. For more information on unacceptable data access restrictions, please see <http://journals.plos.org/plosone/s/data-availability#loc-unacceptable-data-access-restrictions>.

In your revised cover letter, please address the following prompts:

a) If there are ethical or legal restrictions on sharing a de-identified data set, please explain them in detail (e.g., data contain potentially sensitive information, data are owned by a third-party organization, etc.) and who has imposed them (e.g., an ethics committee). Please also provide contact information for a data access committee, ethics committee, or other institutional body to which data requests may be sent.

b) If there are no restrictions, please upload the minimal anonymized data set necessary to replicate your study findings as either Supporting Information files or to a stable, public repository and provide us with the relevant URLs, DOIs, or accession numbers. For a list of acceptable repositories, please see <http://journals.plos.org/plosone/s/data-availability#loc-recommended-repositories>.

We will update your Data Availability statement on your behalf to reflect the information you provide.

>>> Thank you for the information. We have no ethical or legal restriction, so we upload the data set to the FLGSHARE repository. We address this information in the cover letter.

2. Please include your tables as part of your main manuscript and remove the individual files. Please note that supplementary tables (should remain/ be uploaded) as separate "supporting information" files.

>>> We have added our tables in the manuscript.

**Answers to reviewer #1 comments**

**Reviewer #1:**

Dear authors,
I have great interest read your paper.

1. Introduction. Very well referred and up to standards. Given the focus and hunting competition the introduction is weak with respect to that. Please frame the competition better to enlighten the reader.

>>> We have added a paragraph about how the mate competition mediate the trait expression and hormone levels in male Iberian red deer. Also, we comment its implications in costs in terms of stress and immune function, taking into account the mating effort (e.g. testosterone levels) and the investment in sexual traits, such as antlers length and the dark ventral patch size.

2. Study area etc. Please add when in relation to the rut the bulls were shot. Could that - time - explain some of the unexplained variation and that older males had lower concentrations of some compounds? What if older males are active earlier during the rut but shot at the same time or later?

>>> Thank you for your comments. We added the specific date that the samples were collected in each population in the “Study area” epigraph. In all cases, these dates are not far from the rutting season (the most faraway case it is a month from the mating season), so we did not expect that this could have an influence on compounds concentrations between individuals of different age categories. It is worth to highlight that the date of sampling the study populations were not chosen using any criteria, but they were randomly sampled just depending on the hunting activity.

However, we agree with you that older males are active earlier in the rut than subadults or young males. But older males do not cease to be active earlier than these other younger individuals. Hence, we do not expect differences in compounds between age categories due to the same passage of time.

3. Results. OK.
4. Discussion. OK, but what if older males are active earlier in the rut irrespective of level of competition?

>>> Despite it could be true that older males were active in the rut a little bit earlier than subadult and young males, we would expect to find similar temporal pattern of the loss or volatilization of compounds that impregnate the fur in both competition scenarios. However, we found differences in compound profiles between intrasexual competition level at the same category age.

All the best and good luck with your research.

>>> Thank you very much.

**Answers to reviewer #2 comments**

**Reviewer #2:**

Authors studied differences in the presence and concentrations of several chemical scent constituents in the dark ventral patch of Iberian red deer males. They analysed hairs of 84 males originated from two groups of populations related to the harvesting pressure which affected demographic structure of both groups and consequently also caused differences in intraspecific competition, i.e. forming high vs. low male-male sexual competition situations. In these two groups, they studied differences in chemical scent constituents in dark ventral patches of males, which are formed during the rut period and contain volatile compounds that are used in intraspecific communication.

The influences of the age and the dominance of red deer males (and males of some other species) have already been studies several times as is recognized also by the authors of the manuscript; however, the influence of the intensity of the intrasexual competition on the composition of the scent compounds (and hence on the intraspecific communication) has not been studied so far. By finding two different groups of red deer considering their demographic structures and former knowledge on the reproductive efforts of red deer stags in both groups authors managed to find relevant situation in situ allowing also studying of the influence of the male-male sexual competition on the extraction of volatile chemical compounds. They found significant differences in concentrations/proportions of several compounds and managed to provide logical explanations to this variation in relation to differences in intersexual competition among stags of both groups.

I strongly believe that the paper is worth to be published in the PLOS ONE after the minor revision, i.e. after correction of some mistakes that are stated below:

Title: “Iberian male red deer” should be changed either to “Iberian red deer males” or to “male Iberian red deer”. >>> OK, changed
Line 101: please, delete double space after “[25]” >>> OK, changed
Line 138: “estates” instead of “states” >>> OK, changed
Line 139: “in study areas” instead of “in these populations” >>> OK, changed
Line 155: “vs.” instead of “vs” >>> OK, changed
Line 157: add “,” after “competition” >>> OK, changed
Line 161: delete “recently” >>> OK, changed
Line 162: “estates” instead of “states” >>> OK, changed
Line 170: “or” instead of “of” >>> OK, changed
Line 210: delete “,” after “since” >>> OK, changed
Line 228: “0.5%” instead of “0.5 %” >>> OK, changed
Line 244: add “,” after “(5.22%)” >>> OK, changed
Line 275: please, use proper symbols “±”, and use “%” without the space after the number >>> OK, changed
Line 279: please, use proper symbols “±”, and use “%” without the space after the number >>> OK, changed
Line 288: “we compared” instead of “when comparing” >>> OK, changed
Line 313: delete “.” after “above” >>> OK, changed
Line 317: add space after “vs.” >>> OK, changed
Line 319: add “,” after “P = 0.0001)” >>> OK, changed
Line 320: add “, respectively” before “(Fig. 1)” >>> OK, changed
Line 402: please, write “Camelopardalis” in lower case >>> OK, changed
Line 426: delete “, Odocoielus virginatus,” (already used in the line 60, also misspelling) >>> OK, changed
Line 445: delete “(Cervus elaphus hispanicus)” (already used) >>> OK, changed
Line 452: “behavioural” instead of “behavioral” >>> OK, changed

References should be strictly edited according to the PLOS ONE standard; some mistakes are:
L467: “-“ at the end should be “.”; >>> OK, changed
L478: double “.” >>> OK, changed
L479: “:” should not be bolded >>> OK, changed
L481: “-“ instead of “–“ >>> OK, changed
L483: “-“ instead of “–“ >>> OK, changed
L491: double space before title >>> OK, changed
L511: “8” should not be bolded, webpage is mentioned only here >>> OK, changed
L527: double space before “79” >>> OK, changed
L528: change “,” after “Carranza” into the empty space >>> OK, changed
L529: “-“ instead of “–“ >>> OK, changed
L541: “-“ instead of “–“ >>> OK, changed
L580: “-“ instead of “–“ >>> OK, changed
L595: “-“ instead of “–“ >>> OK, changed
L597: “2006” should not be in italic; “-“ instead of “–“ >>> OK, changed
L600: “-“ instead of “–“ >>> OK, changed
L611: without “(4)” >>> OK, changed
L633: “2016” should not be bolded >>> OK, changed
L642: “.” should not be bolded >>> OK, changed

Figure legend:
Line 650: “4-6” instead of “4.6” >>> OK, changed
Line 651: “y:” instead of “y :” >>> OK, changed

Title of the Table 2: “0.5%” instead of “0.5 %” >>> OK, changed

>>> Thanks for your comments and corrections, we are very grateful for it.
